# Synergism between Hydramethylnon and *Metarhizium anisopliae* and Their Influence on the Gut Microbiome of *Blattella germanica* (L.)

**DOI:** 10.3390/insects11080538

**Published:** 2020-08-15

**Authors:** Yu Chao, Mingyue Wang, Wei Dai, Fengyue Dong, Xuejun Wang, Fan Zhang

**Affiliations:** 1 Key Laboratory of Animal Resistance Biology of Shandong Province, College of Life Science, Shandong Normal University, 88 East Wenhua Road, Jinan 250014, China; 17854005019@163.com (Y.C.); ww278661254@126.com (M.W.); dw809283005@163.com (W.D.); dongfy6@163.com (F.D.); 2 Shandong Center for Control and Prevention, 16992 Jingshi Road, Jinan 250014, China

**Keywords:** *Metarhizium anisopliae*, hydramethylnon, synergism, *Blattella germanica*, gut microbiota, biological control

## Abstract

**Simple Summary:**

The widespread use of insecticides has cause extensive resistance in German cockroach (*Blattella germanica*) populations globally. Biological control has the potential to mitigate insecticide resistance, and *Metarhizium anisopliae*, an entomopathogenic fungus, alone and in combination with various insecticides has shown good effects against cockroaches. This experiment compared the cumulative mortality after infecting *B. germanica* with *M. anisopliae* conidia by *per os* infection and topical dorsal infection. To probe the mechanisms that underlie the synergism between *M. anisopliae* and hydramethylnon, we conducted dose-response assays with cockroaches fed combinations of them and characterized the gut microbiome of treated cockroaches. The results showed that the mortality of *per os* infection was lower than that of topical dorsal infection. In addition, the combination of *M. anisopliae* and hydramethylnon had a synergistic effect. The gut microbiome was also altered by hydramethylnon treatment. Therefore, we speculate that one of the mechanism underlying this synergism is that hydramethylnon promotes the survival of *M. anisopliae* in the harsh gut environment and enhances its virulence on German cockroaches by altering the gut microbiome. This may help to develop new types of bio-control glue baits for the control of cockroaches.

**Abstract:**

(1) Background: The widespread use of insecticides has cause extensive resistance in German cockroach (*Blattella germanica*) populations globally. Biological control has the potential to mitigate insecticide resistance, and *Metarhizium anisopliae* (Meschn.) Sorokin, an entomopathogenic fungus, alone and in combination with various insecticides, has shown good effects against cockroaches. (2) Methods: This experiment compared the cumulative mortality after infecting *B. germanica* with *M. anisopliae* conidia by two routes, per os and topical application. To probe the mechanisms that underlie the synergism between *M. anisopliae* and hydramethylnon, we conducted dose–response assays with cockroaches fed combinations of *M. anisopliae* and hydramethylnon and characterized the gut microbiomes of the treated cockroaches. (3) Results: The study showed that the mortality with per os infection was lower than that with topical application. In addition, the combination of *M. anisopliae* and hydramethylnon had a synergistic effect in 16 treatments. The gut microbiome was also altered by hydramethylnon treatment. The abundance of *Parabacteroides* and *Enterococcus* declined with the hydramethylnon and combination treatments, which are known to have anti-inflammatory and antifungal activities. The abundance of *Alistipes*, which is a fungal cell wall component, significantly increased in these treatments. (4) Conclusions: Therefore, we speculate that the major mechanism underlying this synergism is hydramethylnon promoting the survival of *M. anisopliae* in the harsh gut environment and enhancing its virulence for German cockroaches by altering the gut microbiome. This may provide a method for the fight against *B. germanica* and lay the foundation for the development of new baits.

## 1. Introduction

The German cockroach *Blattella germanica* (L.) (Blattaria: Blattellidae) (*B. germanica*), a nuisance pest distributed worldwide, carries a variety of pathogenic microorganisms and eggs of parasites such as cestodes, hookworm and round worms, which can spread diseases and allergens [1,2,3]. Concerning the threat *B. germanica* poses to human health, various measures have been widely applied to control *B. germanica*. Currently, chemical control has become the most important means of controlling *B. germanica* due to its efficiency, low cost and other advantages [3]. However, the extensive use of pesticides, such as pyrethroids, carbamate, neonicotinoids and organofluorine, against *B. germanica* has resulted in the evolution of insecticide resistance, which has become a major challenge for pesticide efficacy [4,5]. Assays indicated that German cockroaches have different levels of resistance to insecticides such as beta-cypermethrin, acephate and carbamate [6,7,8]. There is an urgent need to explore alternative strategies for cockroach control.

Some reagents with great effects have already been developed, and one of them is hydramethylnon [9]. Hydramethylnon is effective in the control of cockroaches and ants [10]. It is a mitochondrial respiratory blocker in insect cells and is a terpenoid [11,12]. It is mainly used as an active ingredient in stomach poison bait, which has relatively mild killing symptoms with the addition of a sputum attractant. Meanwhile, hydramethylnon has been shown to have low toxicity to mammals and cannot cause allergic reactions. Hydramethylnon has been used around the world for more than a decade, and to date, only low resistance has been developed against it [13,14].

Biological control is also very promising for combating cockroaches [2]. In pest management, entomopathogenic fungi, such as *Metarhizium* and *Beauveria* [15,16,17,18,19], are a potential alternative with high biodegradability and specificity and a low likelihood of resistance development [20]. Because entomopathogenic fungi are environmentally friendly and have low virulence for non-target insects, fungus-based microbial source pesticides have been widely used [21,22,23,24,25,26]. Due to the different properties of *Metarhizium anisopliae* (*M. anisopliae*) strains, some of them have shown low virulence for German cockroaches [27,28], and some strains have shown to induce high mortality in cockroaches, which have been deployed in commercial biocontrol products [29]. However, biological agents have limitations such as a long response and slow effect compared with conventional insecticides, limiting their wider use [2]. The combination of entomopathogenic fungi and chemical pesticides provides a great solution for the control of insects. For the control of German cockroaches, a synergistic interaction between boric acid and *M. anisopliae* resulted in great mortality. According to a previous study, the topical application of *M. anisopliae* alone (8.96 × 10^9^ conidia/m^2^) required 28 days to cause 92% cockroach mortality (LT_50_ = 10 days), but in combination with boric acid, *M. anisopliae* conidia dust (8.96 × 10^8^ conidia/m^2^) with either 12.5% (*w*/*w*) boric acid dust or 0.1% (*w*/*v*) boric acid in drinking water killed 100% of the cockroaches in only 8 days (LT_50_ = 5 days) and 10 days (LT_50_ = 6 days), respectively [21]. The cumulative mortality induced by the *M. anisopliae* and β-cypermethrin compounds in the locusts reached 97.8% and 97.3% after 5 days, respectively, which was significantly higher than that in locusts treated with 1 × 10^10^ conidia/mL of *M. anisopliae* [30]. These results showed that *M. anisopliae* has good compatibility with a variety of insecticides.

The gut lumen of German cockroaches contains an abundance of microbes, which are considered to be essential for the growth, development and fertility of the host [3,26,31,32,33]. In addition to producing all of the essential amino acids, various vitamins and other required compounds, these microbes also play important roles in protecting the host insect from the invasion of entomopathogens and boost the immune system of the host [34,35,36,37,38,39,40,41]. Some microbes with anti-entomopathogenic fungal activity contribute to resisting pathogenic fungal infections. For instance, *Lactobacillus* and *Weissella* can produce many antimicrobial agents, such as bacteriocins, adhesion inhibitors and organic acids [33,42]. *Bacteroides* can protect the host insect from pathogens proliferating in the gut by stimulating the immune system of the host, and *Pseudomonas* is characterized by the secretion of versatile secondary metabolites [43,44] and is known for its antifungal activities. The ingestion of entomopathogens also appears to be a rare route of infection and has been reported in a few species of insects [26,45,46,47]. The low efficacy of entomopathogens when ingested matters particularly in the context of delivering conidia in baits, and hydramethylnon has a stomach poisoning effect.

Since *M. anisopliae* has good compatibility with a variety of insecticides and the development level of resistance to hydramethylnon is low, the effect of their combination was evaluated in this study. Because intestinal microbes play important roles in combating insect pathogenic fungi, we conducted high-throughput sequencing of the intestinal microbes of cockroaches to reveal the mechanism of the compounds of *M. anisopliae* and hydramethylnon. This approach may help to provide a promising and effective method for cockroach control.

## 2. Materials and Methods 

### 2.1. Insects

*Blattella germanica* were provided by the Key Laboratory of Animal Resistance Biology of Shandong Province. These cockroaches were cultured in a growth chamber at an appropriate temperature and humidity [27 ± 1 °C, 60 ± 5% relative humidity (RH)] with a 12:12 h light/dark cycle and fed water and rat pellets. The tested insects were adult males of *B. germanica.*

### 2.2. Entomopathogenic Fungi and Fungal Cultivation

The *M. anisopliae* strain EB0732 was isolated from field-collected *Eupolyphaga sunensis* cadavers [19] and preserved by the Key Laboratory of Animal Biology, Shandong Normal University. The entomopathogenic fungal conidia were maintained on potato dextrose agar (PDA) and incubated for 9 days at 28 °C. The growth of the fungi was checked daily, and uncontaminated fungi were transferred to fresh PDA without additional antibiotics to prepare a pure culture. After the growth period, conidia were collected with a sterile metal loop and suspended in sterile phosphate buffered saline (PBS, 3 mM) containing 0.1% (*v*/*v*) Tween 80. The required concentrations of conidial suspensions were determined using a Neubauer hemocytometer (Kanwin Biotechnology Co., Ltd., Shanghai, China).

### 2.3. Hydramethylnon Baits 

To produce baits containing 0.25, 0.50, 0.75 and 1.0% (*w*/*w*) hydramethylnon in rat pellet feed, hydramethylnon was grounded into power, mixed with 2 mL of cooking oil and incorporated into 2 g of rat pellet feed powder (*v*/*w*) to generate 2.5, 5, 7.5 and 10 mg of hydramethylnon, respectively, per gram of semisolid bait. The baits were left to dry at room temperature for 12 h. Cockroaches were provided with fresh treated or untreated baits every 5 days, and the old baits were removed.

### 2.4. Experimental Design of Per Os Infection and Topical Dorsal Infection with M. anisopliae of German Cockroaches

Before the experiment, male German cockroach adults were starved for 24 h. The cockroaches were divided into ten groups of 20 cockroaches. The first five groups were inoculated on the surface of the dorsal cuticle with 2 μL of conidial suspensions of 1 × 10^5^, 1 × 10^6^, 1 × 10^7^, 1 × 10^8^ or 1 × 10^9^ cfu/mL. Conidia (in 2 μL) was applied to the other five groups with a microinjector between the paraglossae (mouthparts); the surface was sterilized with 0.1% mercuric chloride and rinsed three times with sterile water to remove adhering conidia [19]. Sterile PBS (0.1% (*v*/*v*) Tween 80) was used as a negative control. Each concentration was replicated 3 times. After the conidial suspension was added for 24 h, the mortality was recorded every 24 h for 14 days. Only dead cockroaches covered with mycelium were counted as having a fungal infection. Bioassays were monitored daily for 14 days. The data were analyzed by probit analysis with the SPSS 20.0 software.

### 2.5. Experimental Design of M. anisopliae and Hydramethylnon

Before the experiment, male German cockroach adults were starved for 24 h in advance. For bioassays with *M. anisopliae* alone, the cockroaches were divided into four groups of 20, and 1 × 10^5^, 1 × 10^6^, 1 × 10^7^ and 1 × 10^8^ cfu/mL *M. anisopliae* conidial suspensions were added to each group by means of per os infection as indicated in Section 2.4. Each group was replicated 3 times. The bioassays were monitored daily for 14 days. For the bioassays with hydramethylnon baits alone, the cockroaches were divided into four groups of 20, and 0.25, 0.5, 0.75 or 1.0% (*w*/*w*) hydramethylnon bait was given to each group. Each concentration was replicated 3 times. The bioassays were monitored daily for 14 days. Control cockroaches were treated only with PBS solution and pure rat food. For the synergy bioassays, four concentrations of hydramethylnon (0.25, 0.5, 0.75 and 1.0% *w*/*w*) and four concentrations of *M. anisopliae* (1 × 10^5^, 1 × 10^6^, 1 × 10^7^ and 1 × 10^8^ cfu/mL conidial suspensions) were combined in 16 treatments, each of which was replicated 3 times. Each cockroach was fed 2 μL of *M. anisopliae* conidial suspension first; the surface was sterilized with 0.1% mercuric chloride and rinsed three times with sterile water to remove conidia adhering to its external surface. The cockroaches were in groups of 20, hydramethylnon bait was continuously provided, and the old baits were removed. The bioassays were monitored daily for 7 days. The data were subjected to probit analysis using SPSS 20.0 for Windows, and the LT_50_ values were estimated with 95% confidence intervals.

The joint action of the fungus and insecticide was determined using chi-square tests [24]. The 7 day cumulative mortality due to each component of a mixture and the binary mixture was determined empirically from the dosage–mortality curves. The expected values were calculated using the formula O_i_ + O_m_ (1-O_i_), where O_i_ was the empirical mortality caused by insecticides and O_m_ was the empirical mortality caused by *M. anisopliae*. The expected value was then converted into percentage mortality. Therefore, the chi-square values were equal to

(observed% mortality − expected% mortality)^2^/expected% mortality (1)

Significant differences between the treatment combinations according to factorial analysis indicate that there is an interaction between the *M. anisopliae* and insecticide and that the effect observed might be synergistic or antagonistic. By contrast, if there is no significant difference for the *M. anisopliae* plus insecticide combination, it is implied that the effects are additive.

### 2.6. Preparation of Gut Homogenates

German cockroach male adults were divided into 4 groups, with 30 in each group. After 24 h of starvation, the first group was provided with 1% hydramethylnon baits (H), group two was orally fed 1 × 10^8^ cfu/mL of *M. anisopliae* suspension (2 μL) (M), the third group was provided with 1% hydramethylnon baits and 2 μL of *M. anisopliae* suspension at 1 × 10^8^ cfu/mL (HM), and group four was a negative control fed 2 μL of deionized water (C). Each concentration was replicated 3 times. Considering that the characteristics of the gut, physiology and biochemistry of *B. germanica* changed greatly, the cockroaches that were treated for three days were sterilized with 75% ethanol for 90 s and rinsed with sterile water 3 times. Then, the guts were dissected and stored at −80 °C in Eppendorf tubes for further use.

### 2.7. PCR Amplification and Pyrosequencing

Gut homogenates of 120 cockroaches was prepared, and the DNA from the samples was extracted using the K2306 Karroten Microbial Genomic DNA extraction kit (Novoprotein Scientific Inc., Shanghai, China). The V6 variable regions of the 16S rRNA gene were amplified using the primers 515F and 907R (5′-GTGCCAGCMGCCGCGG-3′ and 5′-CCGTCAATTC MTTTRAGTTT-3′, respectively) [3,26]. PCR was conducted in a total volume of 20 μL containing 2 μL of dNTPs (2.5 mM), 4 µL of 5×FastPfu buffer, 10 ng of DNA template, 0.4 µL of FastPfu polymerase (Novoprotein Scientific Inc), 0.8 µL of each primer (0.5 μM) and deionized ultrapure water (to 20 μL). The amplification program was as follows: initial denaturation at 95 °C for 3 min; amplification with 27 cycles of denaturation at 95 °C for 30 s, annealing at 55 °C for 30 s, and extension at 72 °C for 30 s; and a final extension at 72 °C for 10 min. The target PCR product (3 μL) was visualized by electrophoresis on a 2% agarose gel. After the PCR products were purified and quantified, the samples were combined at equal concentrations. Parallel tagged sequencing was performed using MiSeq Sequencing in MAJORBIO based on Solexa Sequencing Technology (Illumina, San Diego, CA, USA).

### 2.8. Bioinformatic and Statistical Analysis

Since the MiSeq sequencing resulted in paired-end sequences, first, the paired reads were spliced to form sequences according to the overlap relations between the paired-end reads; the quality of the reads and the effect of merging were quality controlled and filtered to obtain high-quality reads using the FLASH software (http://ccb.jhu.edu/software/FLASH), with a minimum overlap of 10 bp. Other parameters were left at default settings. The pyrosequencing data were subjected to bioinformatic analysis. Before analysis, the original pyrosequencing data were filtered and optimized using MOTHUR (http://www.mothur.org) and TRIMMOMATIC to obtain valid and trimmed sequences. Chimeric sequences were removed using UCHIME; valid sequences were simplified using the “unique.seqs” command to obtain a unique set of sequences, aligned using the “align.seqs” command and compared to the Bacterial SILVA database (http://www.arb-silva.de). The ”Screen.seqs”, “filter.seqs”, “unique.seqs” and “dist.seqs” commands were used in order. Furthermore, unique sequences were clustered into operational taxonomic units (OTUs), defined at the 97% similarity threshold with MOTHUR and chopseq (MAJORBIO), using MOTHUR and plot rarefaction (MAJORBIO) to carry out rarefaction analysis. According to these results, the community richness (Chao1 and ACE) and diversity indexes (Shannon and Simpson) were estimated using MOTHUR. Community comparison was carried out using the UniFrac Server. A heatmap was generated based on the relative abundance of genera using the R package heatmap followed by principal component analysis (PCA) and nonmetric multidimensional scaling (NMDS) using the R package vegan [48,49].

The results are represented as the mean ± standard error (SE). Statistical analysis was performed by one-way analysis of variance (ANOVA) using SPSS version 20.0 for Windows (IBM, Armonk, NY, USA). A *p-*value less than 0. 05 was considered to be statistically significant. *Blattabacterium* was identified in the results, but the content in each experimental group was less than 0.01%, so it was merged into “others”.

### 2.9. Data Accessibility

All the original data related to the 16S rRNA and metagenomic sequencing in this study are available at Mendeley Data [50]. All the sequences were deposited in the NCBI SRA (Sequence Read Archive) database under the BioProject accession numbers from SRR11668662 to SRR11668673.

## 3. Results

### 3.1. Mortality of Cockroaches with M. anisopliae and Hydramethylnon

**Figure 1:**
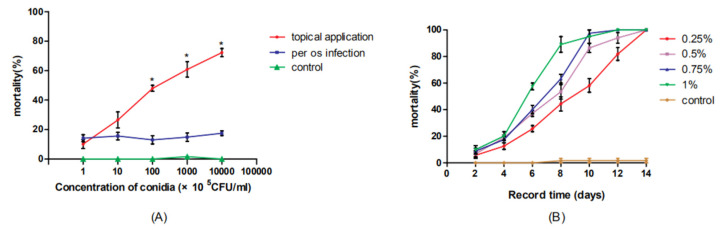
(**A**) The cumulative mortality of *B. germanica* infected with *M. anisopliae* conidia at different concentrations with per os infection and topical application at 14 days postinoculation under laboratory conditions; * indicates that the mortality with topical dorsal infection significantly differs (*p* < 0.05) from that with per os infection. (**B**) The cumulative mortality of *B. germanica* fed with hydramethylnon at different concentrations in baits at 14 days postinoculation under laboratory conditions.

The mortality with per os infection was lower than that with topical dorsal infection, and the difference between the two became increasingly obvious as the concentration of *M. anisopliae* increased (Figure 1A). At a concentration of 1 × 10^9^ cfu/mL, the mortality rate caused by topical dorsal infection was 68.3%, while the per os infection rate was 16.67% (*p* < 0.05). In addition, the mortality with per os infection was not affected by the changing concentrations, but the mortality with topical infection increased in a dose-dependent manner. The cockroaches treated with different concentrations of hydramethylnon exhibited 100% mortality within 14 days. Especially, when they were treated with hydramethylnon at concentrations of 0.75% and 1% *w*/*w*, 100% mortality was achieved within 12 days, respectively (Figure 1B).

### 3.2. Mortality Bioassays and Synergistic Effect Analysis across 16 Treatment Groups

For hydramethylnon treatment, ingested hydramethylnon killed cockroaches in a dose-dependent manner, and the LT_50_ values decreased with increasing concentration (Table 1). At 1.00% *w*/*w* hydramethylnon, the LT_50_ value was 6.6 days, with an LT_50_ of 8.1 days at 0.25% *w*/*w*. 

The effect of *M. anisopliae* delivered by ingestion on cockroaches was significantly lower than that of hydramethylnon (Table 1). No more than half of the insects in the experimental group died within 14 days of being recorded.

**Figure 2:**
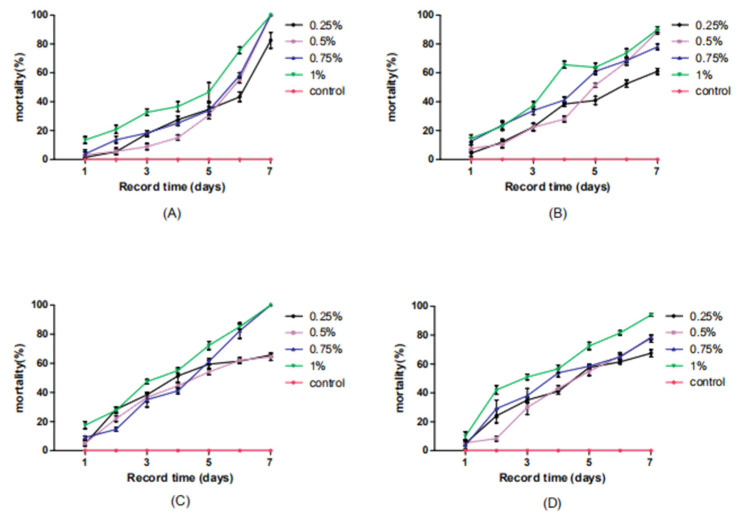
The cumulative mortality of *B. germanica* infected with *M. anisopliae* and hydramethylnon at different concentrations in baits at 7 days postinoculation under laboratory conditions. (**A**) 1 × 10^5^ cfu/mL *M. anisopliae* and 0.25%, 0.5%, 0.75% or 1% hydramethylnon (**B**) 1 × 10^6^ cfu/mL *M. anisopliae* and 0.25%, 0.5%, 0.75% or 1% hydramethylnon (**C**) 1 × 10^7^ cfu/mL *M. anisopliae* and 0.25%, 0.5%, 0.75% or 1% hydramethylnon (**D**) 1 × 10^8^ cfu/mL *M. anisopliae* and 0.25%, 0.5%, 0.75% or 1% hydramethylnon.

The infection results induced by the compound baits were compared (Table 2, Figure 2), and the combination treatments of four hydramethylnon concentrations and four *M. anisopliae* concentrations showed strong synergistic interactions between the two insecticidal agents. All of the treatments, including the lowest concentrations of both (1 × 10^5^ cfu/mL *M. anisopliae* and 0.25% hydramethylnon), resulted in much higher mortality than the additive mortality of hydramethylnon alone and *M. anisopliae* alone (Table 2). Simultaneously, all four combinations of hydramethylnon with 1 × 10^8^ cfu/mL *M. anisopliae* resulted in higher mortality (Table 2). Statistical analysis for the synergistic, additive and antagonistic effects demonstrated strong synergistic interactions between *M. anisopliae* and hydramethylnon in 16 combination treatments. No antagonistic effects were observed in any group. Overall, the use of hydramethylnon strengthened the virulence of *M. anisopliae* for German cockroaches. The control sample showed an extremely low mortality rate of only 3.33% (in 8 days).

### 3.3. Bioinformatic Analysis

After pyrosequencing, a total of 834,715 valid reads and 6458 OTUs were obtained from twelve samples. Each sample contained a different number of phylogenetic OTUs, ranging from 470 to 528 (Table 3), and the maximum number of OTUs reached 404 species in the cockroach gut. Good’s coverage estimations revealed that over 99% of the species were obtained, demonstrating that a sufficient sampling depth was reached for all samples (Table 3). As shown in Supplementary Figure S1, the rarefaction curves tended to approach the saturation plateau and indicated that there were some differences in the total number of OTUs between different samples. In addition, the OTU density in the upper layer was greater than that in the lower parts. In addition, the rarefaction curves revealed that community richness was higher in the control samples (C), but there was no significant difference among all groups.

We estimated the community richness using the ACE and Chao1 indexes and the diversity of the four groups using the Shannon and Simpson indexes. One-way ANOVA was applied to test whether the diversity and richness indexes were significantly different between samples. Both the Shannon and Simpson indexes revealed that the diversity indexes were not significantly different among samples, but the ACE indexes were significantly different (*p* < 0.05) between the H (hydramethylnon), M (*M. anisopliae*), HM (hydramethylnon and *M. anisopliae*) and C samples (Table 3), indicating that the community richness of the four groups was particularly different.

**Figure 3:**
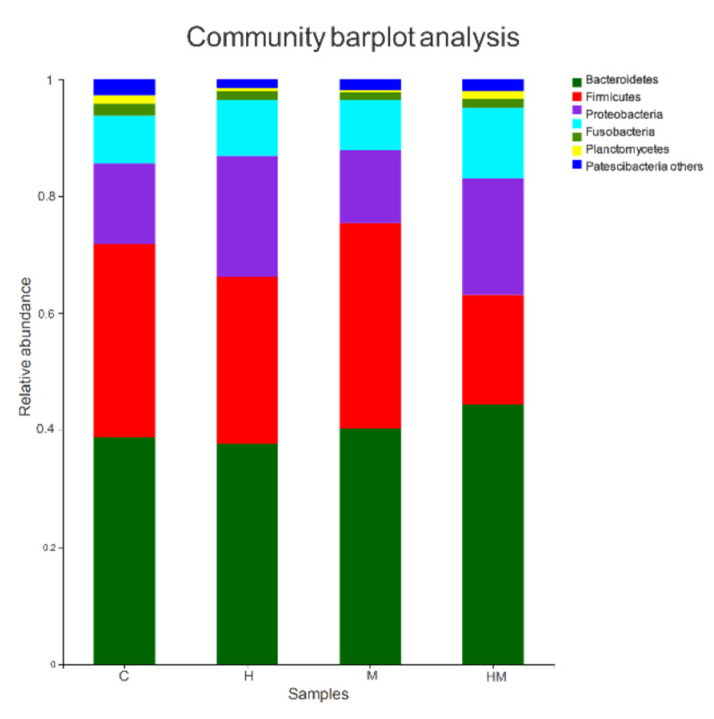
Bacterial composition of the different communities at the phylum level. H, hydramethylnon sample; M, *M. anisopliae* sample; HM, hydramethylnon and *M. anisopliae* sample; C, control sample.

**Figure 4:**
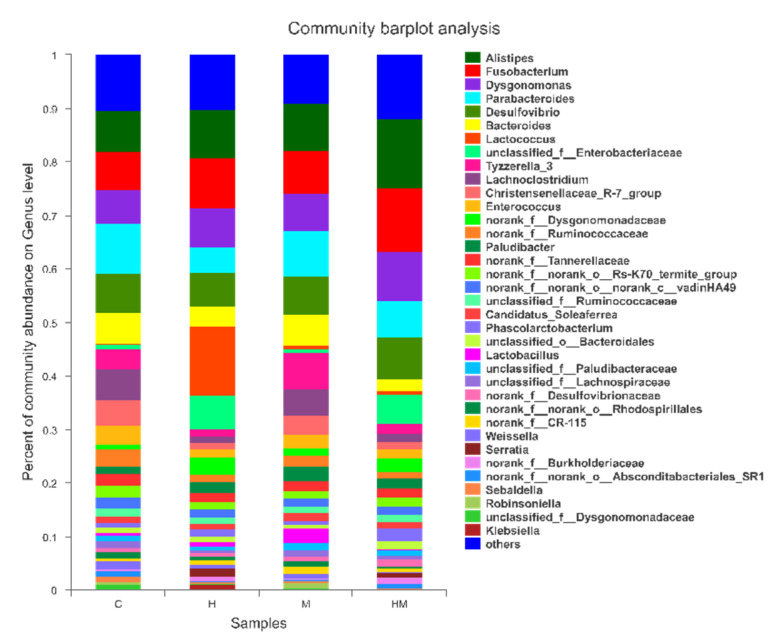
Bacterial composition of the different communities at the genus level. The relative read abundances of different bacterial genera within the different communities are shown. Taxa with an abundance <1% are included in “others.” H, hydramethylnon sample; M, *M. anisopliae* sample; HM, hydramethylnon and *M. anisopliae* sample; C, control sample.

All sequences were classified from the species to phylum level with the Mothur software using the default settings, and 22 phyla and 187 genera were detected (Figure 3 and Figure 4). Sequences that could not be classified were classified as “no rank”. “Others” were taxa with an abundance <1%. The representative sequences at the phylum level are enumerated in Figure 3. Bacteroidetes, Firmicutes, Proteobacteria and Actinobacteria were common to all samples, and Bacteroidetes, Firmicutes and Proteobacteria were the most abundant taxa in all of the samples. The relative abundances of Firmicutes and Proteobacteria were significantly different among the four treatment groups (Firmicutes: HM, 18.70%; H, 38.50%; M, 35.10%; C, 33.01%; *p* < 0.01; Proteobacteria: HM, 19.94%; H, 9.53%; M, 8.55%; C, 13.73%; *p* < 0.05).

At the genus level, the detected OTUs were distributed among 187 different bacterial genera, and the H, M, HM and C samples were composed of 132, 135, 136 and 134 different bacterial genera (Figure 4). The composition of the bacterial genera was similar across the four treatment groups, but their relative abundance varied significantly. In pairwise comparisons of the four treatments at the genus level, there were eight statistically significant differences (Table 4). In the hydramethylnon treatment, the relative abundance of *Parabacteroides* (4.98%) declined significantly relative to that of the control samples (9.29%; *p* < 0.01), as well as *Enterococcus* (1.37% vs. 3.52%, respectively; *p* < 0.05). The relative abundance of *Tyzzerella* in the *M. anisopliae* treatment group was significantly higher (6.63%) than that in the control samples (3.81%; *p* < 0.05). In the *M. anisopliae*–hydramethylnon combination treatment group, the abundance of *Alistipes* (12.98% vs. 7.57%; *p* < 0.01), as well as *Dysgonomonas* (9.33% vs. 6.38%; *p* < 0.05), was significantly increased compared to that in the control samples. However, the abundance of *Enterococcus* was significantly lower in the combination treatment samples than in the control samples (3.52% vs. 1.60%, *p* < 0.05); this was also the case for *Bacteroides* (2.08% vs. 5.78%, *p* < 0.05) in the *M. anisopliae* samples (Figure 4, Table 4).

**Figure 5:**
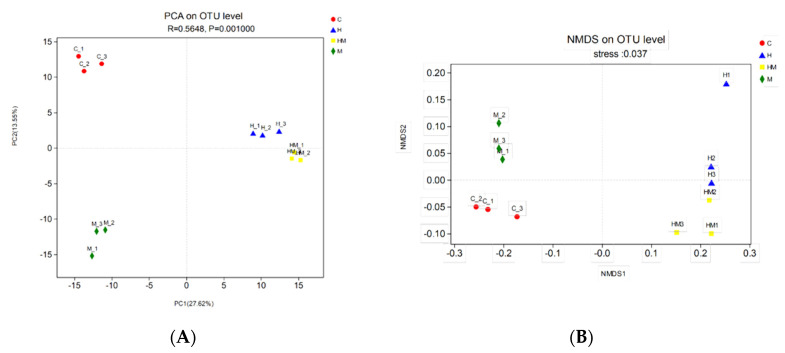
(**A**) Sample sorting analysis. The scatter plot of principal component analysis (PCA) scores shows the similarity of the twelve bacterial communities based on UniFrac distance. Principal components (PCs) 1 and 2 explained 27.62% and 13.55% of the variance, respectively. (**B**) Sample sorting analysis. Nonmetric multidimensional scaling (NMDS) shows the difference among bacterial communities according to Bray–Curtis distance. H, hydramethylnon sample; M, *M. anisopliae* sample; HM, hydramethylnon and *M. anisopliae* sample; C, control sample.

**Figure 6:**
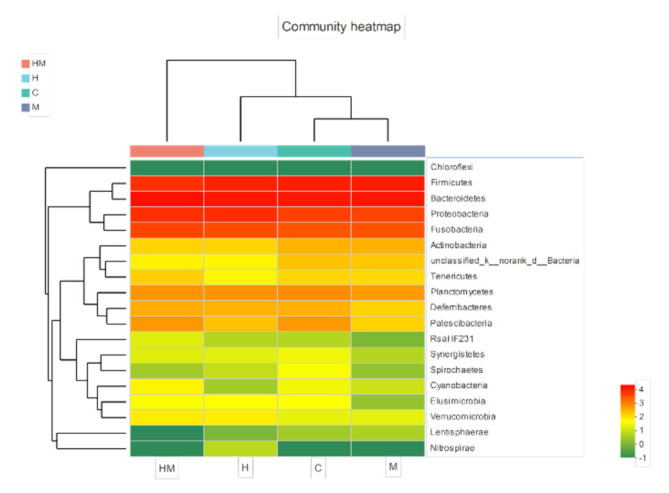
Heatmap of the top 50 most abundant genera in the bacterial communities detected in the 16 samples. Dendrograms for hierarchical cluster analysis grouping genera and sample locations are shown at the left and at the top, respectively. The color scale represents the normalized values of relative abundances transformed by log_10_. Zero values were added as 1 and log_10_ transformed. H, hydramethylnon sample; M, *M. anisopliae* sample; HM, hydramethylnon and *M. anisopliae* sample; C, control sample.

We performed PCA with the weighted UniFrac distance and clustering analysis depending on the pyrosequencing data above. The PCA score plot indicated that the H and HM groups were closely related and grouped to both the left and right of the graph along PC1 and PC2, which explained 27.62% and 13.55% of the variance, respectively. In addition, the C and M groups were separated from H and HM along PC1 and PC2, but they were close to each other along PC1. Overall, the two PCA axes explained 41.17% of the variation between the different communities (Figure 5A). The NMDS analysis, according to the Bray–Curtis distance, also confirmed that bacterial communities in the H and HM samples were significantly different from those in either the C or M samples (Figure 5B). The heat map graphically shows that, at the genus level, the compositions of the bacterial communities in the H and HM groups were more similar than those in the other groups (Figure 6).

## 4. Discussion

Our study shows that the *M. anisopliae* infection rate with per os infection is lower than that with topical dorsal infection (Figure 1A). For the entomopathogenic fungi, the infection cycle can usually be divided into five stages: adhesion to the cuticle, germination, penetration through the exoskeleton, host tissue colonization and sporulation [51,52,53]. *M. anisopliae* could produce some specific degrading enzymes to penetrate the cuticle of the insect and adapt to the highly permeable environment of the host’s hemolymph based on a MOS1 osmosensor to combat the insect immune system in long-term evolution [54,55,56]. This allows the fungus, to a certain extent, to escape the immune mechanism of the host hemocoel and the production of hyphal bodies [57]. These mechanisms help conidia to successfully invade the hemocoel of cockroaches by topical dorsal infection. For the per os infection, we concluded that the characteristics of the digestive tract environment in cockroaches, including the digestive enzymes, pH, low O_2_ and high osmotic pressure, are not conducive to the germination and reproduction of conidia, and the symbiotic bacteria in gut may also produce antimicrobial peptides and other substances to inhibit and kill conidia [27,33]. As for there being no dose response in per os infection, this may be due to most conidia remaining too short in the digestive tract to successfully infect cockroaches (Figure 1A).

In this study, we found that the greatest mortality occurred with the lowest *M. anisopliae* dosage, but lower mortality was seen with a higher *M. anisopliae* dosage (Table 2). We speculated that the high concentration of *M. anisopliae* conidia would affect the taste of the bait of hydramethylnon, reducing the palatability of the bait and thus affecting the appetite of the insects, thereby reducing the intake of the bait, leading to the decrease in the mortality rate. Thus, we suspect that is why increasing the *M. anisopliae* and hydramethylnon concentration did not cause a linear increase in mortality. In addition, *M. anisopliae* can infect insects through body wall contact. If *M. anisopliae* is added to the bait, it would be likely to come into contact with the body of the insect, which could cause contact-associated mortality and confound the experimental results.

Multiple studies have shown synergistic effects between *M. anisopliae* and various chemical insecticides, such as cypermethrin and boric acid. The mixture of *M. anisopliae* and cypermethrin can assist the infection of *M. anisopliae* spores by reducing the expression of hemolymph protein [58]. Boric acid, also known as a stomach toxin, had a high insecticidal effect in complex with *M. anisopliae* [21,28]. Based on previous studies, it was thought that the synergistic mechanism involved boric acid leading to damage to gut epithelial cells or gut cuticles, thus facilitating the penetration of *M. anisopliae*. There may have been a similar mechanism in the experiments we performed [59,60]. Meanwhile, several studies have shown insecticidal impacts on the gut microbiome of cockroaches, such as *M. anisopliae* and beta-cypermethrin [3,26]. Thus, the recognition that the gut microbiome is a significant participant in insecticide toxicology also prompted us to compare the microbiomes of cockroaches exposed to various hydramethylnon and *M. anisopliae* combinations. Thus, we analyzed the gut microbiota of four different cockroach treatment groups and performed Solexa high-throughput sequencing.

According to the results of the experiments, the structure and composition of the intestinal microbiomes of cockroaches in our study are roughly the same as those in previous studies [3,26], but the use of hydramethylnon would cause additional changes. The groups treated with hydramethylnon and *M. anisopliae* combined with hydramethylnon had some differences from the others (Table 4). Hydramethylnon ingestion decreased the relative abundance of *Parabacteroides, Tyzzerella* and *Enterococcus* compared with that in the control samples. The relative abundance of *Tyzzerella* and *Enterococcus* was also lower in the combined hydramethylnon–*M. anisopliae* treatment group, implying that hydramethylnon was responsible for these changes. All the bacterial taxa are known to have anti-inflammatory effects and protect the host from fungal invasion [61]. *Enterococcus* has antifungal activity against several fungi, including *Candida albicans*, *Debaryomyces hansenii* and *Penicillium roqueforti*. It also produces three bacteriocins, EntV, durancin A5-11a and durancin A5-11b, which have similar antimicrobial properties [62,63]. Although we do not know the role of *Tyzzerella* in the guts of cockroaches, activity tests of the purified proteins revealed that *Tyzzerella* was able to hydrolyze the disaccharide unit from Galβ1-3GalNAc-α-*p*NP [64]. *Parabacteroides* is involved in the degradation of complex organic matter, providing amino acids and cofactors for the nutrition of the cockroach host and producing many antimicrobial agents such as organic acids, bacteriocins and adhesion inhibitors [65,66,67]. Considering that hydramethylnon inhibits both *Parabacteria* and *Enterococci*, we speculate that it assists *M. anisopliae* in penetrating the intestinal wall of German cockroaches. By contrast, the relative abundance of *Alistipes* and *Dysgonomonas* significantly increased in the hydramethylnon–*M. anisopliae* treatment group. The genus *Alistipes* resembles the *Bacteroides fragilis* group and appears to be involved in carbohydrate metabolism. It is of particular interest that *Alistipes* species can hydrolyze chitin, which is a fungal cell wall component [34,68]. *Dysgonomonas* is a dominant bacterial genus in the intestinal tract of many insects. It can ferment sucrose and glucose to produce lactic acid, acetic acid, propionate, etc. [69]. Accordingly, it can be surmised that hydramethylnon can enhance the infection effect of *M. anisopliae* by causing a nutritional imbalance in cockroach intestines.

In general, hydramethylnon has a stomach poisoning effect, and it can destroy the intestinal flora [70] and the peritrophic membranes of lepidopteran insects, increasing the chances of *M. anisopliae* infection in the digestive tract. Furthermore, hydramethylnon can be absorbed by cockroaches and acts on the mitochondria, destroying cell respiration, thereby inhibiting the metabolic system of the cockroach. Meanwhile, hydramethylnon is a type of respiratory chain inhibitor that can inhibit complex III activity to block ATP production [12,71]. Hydramethylnon is widely used in various insects and has been shown to be effective [72]. Accordingly, the combination of hydramethylnon and *M. anisopliae* proved to be an efficient insecticide through different insecticidal mechanisms, and the use of hydramethylnon can greatly enhance the insecticidal effect of *M. anisopliae*. Overall, our results demonstrate that hydramethylnon and fungal conidia can be used in combination to enhance efficacy, providing safer and effective methods of cockroach control.

Resistance is an obstacle in cockroach control, and the combination of chemical agents and biological agents is an important research direction for managing insecticide resistance [21,28,30,58]. A variety of studies have shown the combination of the two can delay the emergence of insect resistance and improve insecticidal effects [21,28,30,58]. The results of the virulence bioassay in our experiment proved that there is a synergistic effect between *M. anisopliae* and hydramethylnon against *B. germanica*, and the compound is expected to play a beneficial role in practical applications.

## Figures and Tables

**Table 1:** Mortality of *B. germanica* adult males fed various concentrations of *M. anisopliae* and various concentrations of hydramethylnon.

Treatment	Concentration	Mortality (%)	LT_50_ (95% CI)	X^2^	Slope	*n*
Ma	1 × 10^5^ cfu/mL	6 ± 0.63	NA	NA	NA	60
	1 × 10^6^ cfu/mL	8 ± 1.07	NA	NA	NA	60
	1 × 10^7^ cfu/mL	9 ± 0.00	NA	NA	NA	60
	1 × 10^8^ cfu/mL	9 ± 2.28	NA	NA	NA	69
Hy	0.25% *w*/*w*	36 ± 2.49	8.1(6.9–9.2)	0.6079	0.1726	60
	0.50% *w*/*w*	48 ± 1.11	7.4(6.7–7.6)	1.4210	0.1784	60
	0.75% *w*/*w*	49 ± 2.99	7.2(6.9–7.9)	2.3606	0.1774	60
	1.00% *w*/*w*	71 ± 4.66	6.6(6.1–7.2)	0.4526	0.1690	60
Control	--	0	NA	NA	NA	60

Ma, *M. anisopliae*; Hy, hydramethylnon; the mortality was at Day 7; LT_50_ = days until 50% mortality occurred; CI = 95% confidence interval for LT_50_; NA = not applicable (mortality did not reach 50%).

**Table 2:** Bioassays assessing the interactive effects of combination treatments of *M. anisopliae* and hydramethylnon on adult male German cockroaches.

Treatment		% Mortality ± SD	LT_50_ (95% CI) d	Effect	X^2^	*n*
Ma (cfu·mL^−1^)	Hy (%)					
1 × 10^5^	0.25	93 ± 5.77	6.4 (5.7–7.2)	Synergistic	70.93	60
	0.50	100 ± 0.00	5.4 (5.3–5.5)	Synergistic	46.74	60
	0.75	100 ± 0.00	5.4 (4.9–5.2)	Synergistic	44.15	60
	1.00	100 ± 0.00	4.7 (4.6–4.8)	Synergistic	10.22	60
1 × 10^6^	0.25	63 ± 3.67	5.7 (5.3–6.1)	Synergistic	11.64	60
	0.50	90 ± 1.81	5.1 (4.3–5.9)	Synergistic	27.45	60
	0.75	87 ± 9.53	4.9 (4.7–5.0)	Synergistic	21.68	60
	1.00	90 ± 6.93	4.2 (3.6–4.7)	Synergistic	4.26	60
1 × 10^7^	0.25	65 ± 3.88	5.3 (4.8–5.8)	Synergistic	12.93	60
	0.50	67 ± 5.32	4.5 (4.0–4.9)	Synergistic	3.89	60
	0.75	100 ± 0.00	4.2 (3.5–4.9)	Synergistic	40.19	60
	1.00	100 ± 0.00	4.5 (4.2–4.9)	Synergistic	9.46	60
1 × 10^8^	0.25	70 ± 6.73	4.5 (4.0–4.9)	Synergistic	19.10	60
	0.50	80 ± 2.11	4.5 (3.9–5.0)	Synergistic	14.17	60
	0.75	77 ± 6.48	4.2 (3.2–5.3)	Synergistic	10.23	60
	1.00	93 ± 4.52	4.0 (3.5–4.9)	Synergistic	5.11	60

Hy+Ma, the combination of *M. anisopliae* and hydramethylnon; LT_50_ = days until 50% mortality occurred; CI = 95% confidence interval for LT_50_; chi-square values calculated using the formula (observed % mortality − expected % mortality) ^2^/expected % mortality (X^2^ = 3.84, df = 1, α = 0.05).

**Table 3:** Richness and diversity indexes relative to each gut sample (operational taxonomic unit (OTU) cut-off of 0.03).

				Alpha Diversity			
Sample	Threshold	Coverage	Number of OTUs	Shannon	Simpson	Ace	Chao
H_1	0.03	0.998868	490	3.905918	0.096218	521.9473	515.5185
H_2	0.03	0.998664	496	4.698616	0.021041	533.7328	555.0323
H_3	0.03	0.998909	506	4.659649	0.023202	540.7188	557.2069
M_1	0.03	0.998635	518	4.827646	0.017409	568.0368	577.7568
M_2	0.03	0.998659	501	4.72536	0.019885	536.879	530.2041
M_3	0.03	0.998847	493	4.593136	0.027867	521.5302	533.8333
HM_1	0.03	0.998967	503	4.638124	0.028481	548.5247	558.4545
HM_2	0.03	0.998791	497	4.707876	0.025847	524.4121	540.9655
HM_3	0.03	0.998913	470	4.697442	0.020668	497.2496	503.3871
C_1	0.03	0.998601	523	4.853477	0.015883	565.2743	565.9773
C_2	0.03	0.998916	493	4.689701	0.020989	520.4105	528.25
C_3	0.03	0.99859	528	4.629903	0.026876	567.794	571.1707
*p-*value			0.063069	0.3634	0.304064	0.015943	0.200845

OTUs were defined at the 97% similarity level (the threshold is 0.03); H, hydramethylnon sample; M, *M. anisopliae* sample; HM, hydramethylnon and *M. anisopliae* sample; C, control sample; *p* < 0.05 is considered to indicate a significant difference.

**Table 4:** *p*-Values for relative abundance comparison between different treatments.

Genus	HM			H		M
	H	Ma	C	Ma	C	C
***Alistipes***	+0.0018 **	+0.0023 **	+0.0003 ***	+0.6159	+0.0596	+0.1587
***Fusobacterium***	−0.2478	−0.1879	−0.1556	−0.5388	−0.3774	−0.7121
***Parabacteroides***	−0.4078	+0.5644	+0.3913	+0.0084 **	+0.0016 **	+0.4692
***Dysgonomonas***	−0.0643	−0.0004 ***	−0.0207 *	−0.5971	−0.5136	−0.7123
***Desulfovibrio***	−0.1868	−0.6274	−0.6464	+0.2918	+0.4321	−0.945
***Bacteroides***	+0.1495	+0.0448 *	+0.0446 *	+0.1813	+0.1739	+0.9564
***Enterococcus***	−0.6811	+0.0931	+0.0145 *	+0.0097 **	+0.018 *	+0.0951
***Tyzzerella***	−0.2728	+0.0049 **	+0.0049 **	+0.0017 **	+0.0344 *	−0.0427 *

H, hydramethylnon sample; M, *M. anisopliae* sample; HM, hydramethylnon and *M. anisopliae* sample; C, control sample. The sample in the first row is compared with the sample in the second row; an increase or decrease in the abundance of a genus is shownwith “+” and “−“, respectively. * *p* < 0.05, ** *p* < 0.01, *** *p* < 0.001 (one-way ANOVA).

## Supplementary Materials

The following are available online at https://www.mdpi.com/2075-4450/11/8/538/s1. Figure S1: Rarefaction analysis of the different samples. Rarefaction curves of OTUs clustered at the 97% phylotype similarity level. Sobs represents the observed number of species. H, hydramethylnon sample; M, M. anisopliae sample; HM, hydramethylnon and M. anisopliae sample; C, control sample.
