# Supplementary material for: Synergism between Hydramethylnon and Metarhizium anisopliae and Their Influence on the Gut Microbiome of Blattella germanica (L.)
Source: Insects. 2020 Aug 15;11(8):538. doi: 10.3390/insects11080538 (PMC7469186; doi:10.3390/insects11080538)
Supplement: Supplementary file 1 [file insects-11-00538-s001.pdf]

Supplementary

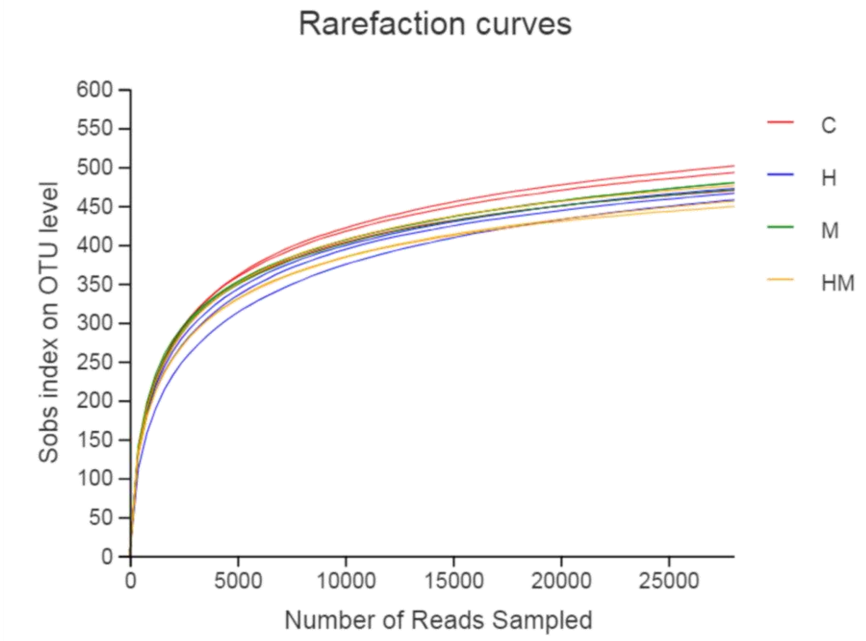

Figure 1. Rarefaction analysis of the different samples. Rarefaction curves of OTUs clustered at the 97% phylotype similarity level. Sobs represents the observed number of species. H, hydramethylnon sample; M, *M. anisopliae* sample; HM, hydramethylnon and *M. anisopliae* sample; C, control sample.
